# Multisession transcranial direct current stimulation and memory-associated cognitive functions in healthy young adults

**DOI:** 10.3389/fnhum.2025.1612308

**Published:** 2025-08-29

**Authors:** Daria Kukuła, Monika Wiłkość-Dębczyńska, Anna Rasmus

**Affiliations:** Faculty of Psychology, Kazimierz Wielki University, Bydgoszcz, Poland

**Keywords:** transcranial direct current stimulation, cognitive functions, memory, neuroplasticity, young adults

## Abstract

Research suggests that cognitive ability is an important predictor of life outcomes, health, and mortality. Cognitive functions develop at different rates depending on age, and human development involves individual and multidirectional changes, which are plastic processes that last throughout life. Memory, a complex and multidimensional cognitive area, comprises various systems that work together to encode, store, and retrieve information. Working memory plays a key role in processing the information required for a current activity. In recent years, non-invasive brain stimulation techniques such as tDCS have attracted growing attention from researchers. tDCS modulates cortical activity via low-intensity current, inducing neuronal membrane polarization. This study aimed to assess the effect of tDCS on memory function in healthy individuals in early adulthood. The sample consisted of 90 volunteers aged 20–35 years which were divided into three groups: experimental, active control, and passive control. Cognitive functions were assessed using the Digit Span Test from the WAIS-R(PL), Berg’s Card Sorting Test (BCST), and the Tower of London Test (TOL). The results indicated that tDCS contributed to improvements in working memory, particularly in the auditory-verbal memory span, the effectiveness of maintaining an intended plan in memory, and the ability to remember and coordinate different components of the task. No significant changes were observed in the digit span backward measurement. This study has limitations, including the absence of long-term follow-up and lack of physiological measures such as qEEG. Nonetheless, the findings suggest that tDCS may be a promising method for enhancing memory-related cognitive functions in early adulthood.

## Introduction

Research shows that cognitive ability is a strong predictor of important life outcomes such as health and longevity. Cognitive functions develop along different trajectories, influenced by age and other factors. Human development is not linear or uniform, but involves individual, multidirectional changes with both gains and losses throughout life (Flensborg-Madsen et al., 2020; Mashburn et al., 2023).

Memory is a complex, multidimensional cognitive domain made up of interconnected systems for encoding, storing, and retrieving information (Bowman and Zeithamova, 2018; Kolb and Whishaw, 2003; Pąchalska et al., 2020). A fundamental component, working memory (WM), allows temporary storage and manipulation of information, supporting goal-directed behavior and executive functions (Angelopoulou and Drigas, 2021; Baddeley and Hitch, 1974; Fuster, 2001).

Efforts to improve cognitive functions continue across both healthy and clinical populations. Traditional methods such as cognitive training can be time-consuming and less engaging, prompting interest in more scalable, efficient approaches. In this context, non-invasive brain stimulation techniques—particularly transcranial direct current stimulation (tDCS)—have gained attention as promising tools for cognitive enhancement (Habich et al., 2021; Zimerman and Hummel, 2010). In contrast to transcranial magnetic stimulation (TMS), tDCS does not trigger neural activity directly but modulates cortical excitability by subtly altering neuronal membrane potentials (Stagg et al., 2009; Woods et al., 2016).

Despite the long history of tDCS (Kropotov, 2006; Sarmiento et al., 2016), it remains an experimental method (Budzisz, 2017; Kukuła et al., 2020; Pąchalska et al., 2020; Woods et al., 2016). Nevertheless, it is widely viewed as safe, with minimal risk when used within standard parameters (Fregni et al., 2005). Common adverse effects, such as skin redness, are mild and transient. tDCS employs low current (1–2 mA) to modulate cortical excitability by altering neuronal membrane polarization, thereby increasing or decreasing brain activity (Budzisz, 2017; Dedoncker et al., 2016; Fertonani et al., 2011; Philip et al., 2017; Yamada and Sumiyoshi, 2021; Woods et al., 2016).

Although the exact mechanisms of tDCS are not fully understood, research suggests that it enhances neuroplasticity (Ghanavati et al., 2022; Mohammadi, 2016; Radman et al., 2009). Anodal stimulation increases cortical excitability via depolarization, while cathodal stimulation reduces it through hyperpolarization (Nitsche and Paulus, 2000). Although the exact mechanisms underlying the therapeutic effects of tDCS are still not fully understood, researchers have proposed that anodal tDCS induces plasticity similar to long-term potentiation (LTP) and long-term depression (LTD) (Ghanavati et al., 2022; Nitsche et al., 2008; Radman et al., 2009). It is assumed that tDCS can cause changes in the inhibitory gamma-aminobutyric acid (GABAergic) systems, which play a critical role in improving neuroplasticity (Nitsche et al., 2003). Furthermore, magnetic resonance spectroscopy showed that anodal tDCS stimulation reduces GABA concentration (Stagg et al., 2009). Fritsch et al. (2010) demonstrated that anodal tDCS applied to the motor cortex (M1) induces long-lasting potentiation that is polarity-specific and NMDA receptor-dependent, and requires coupling of stimulation to repetitive low-frequency synaptic activation (LFS). This interaction increases the secretion of brain-derived neurotrophic factor (BDNF) and activates receptor kinase B (TrkB). These results suggest that BDNF is a key mediator of the tDCS-induced aftereffects. Several studies have also suggested that anodal stimulation of the left dorsolateral prefrontal cortex (with a current intensity of 2 mA) improves memory performance, including working memory (Fregni et al., 2005; Iyer et al., 2005).

Cognitive stimulation in young adults aims to enhance mental capacity, supporting daily and professional demands while also building cognitive reserve (Negash et al., 2013; Stern, 2012). Although tDCS has shown potential benefits in areas such as working memory, attention, learning, and decision-making (for example, Boudewyn et al., 2020; Flöel, 2014; Hauser et al., 2013; Hoy et al., 2013; Imburgio and Orr, 2018; Javadi and Cheng, 2013; Minati et al., 2012; Ohn et al., 2008; Roy et al., 2015; Ruf et al., 2017), evidence in healthy individuals remains mixed. The meta-analytic review by Meduso et al. (2016) revealed a small but significant effect of left DLPFC stimulation coupled with WM training. However, some meta-analyses and controlled studies have reported limited or inconsistent effects, particularly when considering variability in stimulation parameters, individual differences in neuroanatomy, and cognitive baselines (Horvath et al., 2015; Medina and Cason, 2017). The impact of placebo and expectation effects is also increasingly being acknowledged as a confounding factor (Rabipour et al., 2018).

Despite these concerns, the potential of multisession tDCS to induce cumulative effects and more robust cognitive enhancement remains an open question. This study aimed to investigate whether multiple sessions of anodal tDCS in the left DLPFC can improve working memory performance in healthy young adults using both active and passive control groups to help isolate stimulation-specific effects.

## Materials and methods

### Participants

Ninety healthy individuals in early adulthood, aged 20–35 years (*M* = 27.94 years; *SD* = 3.92), qualified for the study. All participants provided written informed consent and reported no history of neurological or psychiatric disorder. Participants were assigned to one of three groups: 1. Experimental, receiving anodal tDCS in the left DLPFC, (EG; *n* = 30); 2. Active control (placebo), receiving sham stimulation (AC; *n* = 30), and 3. Passive control with no stimulation (PC; *n* = 30). Group equivalence was established for sex and age variables. Inclusion criteria included: early adulthood and right-handedness. Exclusion criteria included: metal implants in the cranial region, history of epilepsy or neurological disorders, presence of implanted medical devices (e.g., pacemaker, cochlear implant), history of head trauma or central nervous system damage (e.g., sensory disturbances, spasticity, paresis), ongoing cancer treatment, pregnancy, or prior experience with tDCS. Table 1 shows the characteristics of the study groups.

**Table 1:** Characteristics of the participants in the studied groups.

Number of people surveyed	Experimental group		Active control group		Passive control group	
	*N*		*N*		*N*	
	30		30		30	
Age	*M*	*SD*	*M*	*SD*	*M*	*SD*
	27.33	4.28	28.03	3.85	27.93	3.96
	*Min.*	*Max.*	*Min.*	*Max.*	*Min.*	*Max.*
	21	35	21	35	21	35
Sex	*n*	%	*N*	%	*n*	%
Women	15	50	15	50	15	50
Men	15	50	15	50	15	50
Education	*n*	%	*N*	%	*n*	%
Secondary	6	20	5	16.67	3	10
Higher	24	80	25	83.33	27	90
Number of years of study	*M*	*SD*	*M*	*SD*	*M*	*SD*
	16.10	1.44	15.68	1.28	15.5	1.22

Education: secondary - refers to the completion of high school; higher - refers to the attainment of a bachelor’s or master’s degree.

In the EG group, the mean age was 27.33 years (SD = 4.28), in the AC group 28.03 years (SD = 3.85), and 27.93 years (SD = 3.96) in the PC group. Each group included an equal number of women and men (15 women and 15 men). Most respondents had a higher level of education. There were 24 (80%) participants in the active group, 25 (83.33%) in the active control group, and 27 (90%) in the passive control group. The remaining individuals had secondary education. The mean number of years of education in the groups was similar, i.e., 16.10 years (SD = 1.44) in the GE group, 15.68 years (SD = 1.28) in the AC group and 15.5 years (SD = 1.22) in the PC group.

### Cognitive assessment

***The Digit Span Test from WAIS-R(PL)*** examines the short-term verbal memory span (digit span forward) and working memory (digits backward) (Kotapka-Minc, 2007; Wambach et al., 2011). The number of correct repetitions was measured. The task consists of two parts: forward digit repetition, in which the subject is asked to repeat ascending ranges of digits in the order in which they were presented by the researcher, and backward digit repetition, in which the subject is asked to repeat ascending ranges of digits in the reverse order from which they were presented (Brzeziński and Wechsler, 2011; Wiejak and Krasowicz-Kupis, 2011). The study includes the number of correct repetitions in the forward digit span task as a measure of auditory-verbal memory span and the number of correct repetitions of digits in the backward digit span task, which reflects the ability to maintain, reverse and manipulate verbal information. Both measures are indicators of the efficiency of manipulating information in the working memory and the ability to reorganize newly remembered content.

***Berg’s Card Sorting Test* (BCST)** is the Psychology Experiment Building Language (PEBL) version of *the Wisconsin Card Sorting Test* (WCST). This method was developed by Berg at the University of Wisconsin (Berg, 1948). The test is used to measure executive functions, which are understood as functions that supervise, control, and direct human cognitive activity (Jodzio, 2017). In the test, participants sort cards of different colors, symbols, and numbers into four piles. Through trial and error, they determine the classification principle. Once a consistent correct fit is achieved, the rule is changed. In the computer version, the entire process is automated, which saves time and makes the counting of points easier (Piper et al., 2015). In this study, an indicator expressed as the percentage of correctly performed movements in the BCST reflected the effectiveness of remembering changing rules and adapting to new conditions.

***Tower of London* Test (TOL)** from PEBL assesses executive functions, with particular emphasis on the ability to plan and predict. The essence of tower testing, including the Tower of London Test, is to find the best solution by dividing the entire activity – the final goal – into smaller activities or intermediate tasks. At the same time, the person performing the task must follow strictly established rules and a specific order (Shallice, 1982). The ratio of movements performed to the minimum number of movements was analyzed, which is an indicator of the ability to remember and manipulate information effectively. It assesses whether the tested person can effectively store and manage information in working memory as well as make decisions effectively in the context of planning and prediction.

### tDCS protocol

Stimulation was performed using an 8-channel Starstim tES device with *Neuroelectrics Instrument Controller* management software from Neuroelectrics. Round sponge electrodes with an area of 25 cm^2^ each were used for stimulation. The study used a montage: anodal stimulation in the left dorsolateral prefrontal cortex and cathodal stimulation in the right supraorbital region (F3 and Fp2, respectively, according to the 10–20 EEG system). The dose of electric charge administered during one meeting in the experimental group was 3640.00 mC. Each subject underwent two assessments of cognitive functioning before and after participating in the stimulation cycle. The first measurement was performed 1–3 days before the first stimulation, whereas the second measurement was performed 1–3 days after the completion of the cycle. The experimental group participated in ten 30-min tDCS stimulation sessions during which a current of 2 mA was delivered, with a rise and fall period of 20 s. The electrode assemblies were as follows: anode, F3; cathode, Fp2. Round sponge electrodes with surface areas of 25 cm2 were used. The AC group also attended 10 meetings, during which they participated in a cycle whose procedure was identical to that of the GE group. The only difference was that the subjects received the increasing current only during the first 20 s, so that they were unaware of which group they had been assigned to. The aim was to ensure that the conditions in the PC group were as close as possible to the active stimulation conditions, so that participants could not recognize which group they were in. The inclusion of AC group in the study allowed us to conclude that the changes observed in the experimental group resulted from the impact of the experimental factor and not from the placebo effect (Boot et al., 2013). In the PC group, cognitive functioning was assessed twice with an interval of 14–16 days. Participants in this group did not receive any form of tDCS or any other type of active intervention.

## Results

To test whether tDCS in the F3 anode and Fp2 cathode montage affects memory in healthy young adulthood, a series of 2 (measurement: before and after) × 3 (group: experimental, active control, passive control) mixed-design ANOVAs were performed. The within-subjects factor was the dual-memory measure, and the between-subjects factor was the group.

Table 2 presents the test results, while Tables 3, 4 present the descriptive statistics for the effects obtained.

**Table 2:** Differences in memory depending on the research condition.

Within-subject factor	*F* (1,87)	*p*	η^2^p	Differences
Repeating numbers forward	23.67	**<0.001**	0.22	P1 < P2
Repeating numbers backwards	1.02	0.316	0.01	-
BCST: Correct movements	18.18	**<0.001**	0.17	P1 < P2
TOL: ratio of moves made/minimum number of moves	81.79	**<0.001**	0.49	P1 > P2

Between-subject factor	*F* (2,87)	*p*	η^2^p	Differences: *post hoc* tests
Repeating numbers forward	4.08	**0.020**	0.07	PC < EG
Repeating numbers backwards	4.34	**0.016**	0.09	PC < EG
CBT: Memory span	0.17	0.846	<0.01	-
BCST: Correct movements	3.73	**0.028**	0.08	PC < EG
TOL: Ratio of moves made/minimum number of moves	0.84	0.436	0.02	-

Interaction	*F* (2.87)	*p*	η^2^p	*Post-hoc* tests	
				Differences within groups	Differences between groups
Repeating numbers forward*Group	5.48	**0.006**	0.11	EG, AC: P1 < P2	P2: EG > AC, PC
Repeating numbers backwards*Group	1.78	0.175	0.04	-	-
BCST: Correct movements*Group	36.34	**<0.001**	0.46	EG: P1 < P2	P2: EG > AC, PC
TOL: ratio of moves performed/minimum number of moves*Group	10.72	**<0.001**	0.20	EG, AC, PC: P1 > P2	P2: EG > AC, PC

P, measurement; EG, experimental group; AC, active control group; PC, passive control group. The table presents significant differences at the level of *p* < 0.05. In the case of *post hoc* tests, comparisons were made with the Bonferroni correction.

Bold values indicate statistically significant results (*p* < 0.05).

**Table 3:** Descriptive statistics for digit span test.

Group	Measurement	Forward digit span		Backward digit span	
		*M*	SD*	*M*	SD*
EG	P1	9.27	1.36	7.40	1.33
	P2	10.00	1.08	7.63	1.13
	Total	9.63	0.25	7.52	0.24
AC	P1	8.73	1.70	6.77	1.36
	P2	9.13	1.41	6.80	1.27
	Total	8.93	0.25	6.78	0.24
PC	P1	8.60	1.54	6.60	1.52
	P2	8.67	1.49	6.53	1.43
	Total	8.63	0.25	6.57	0.24
Total	P1	8.87	1.55	6.92	1.43
	P2	9.27	1.44	6.99	1.35

P, measurement; EG, experimental group; AC, active control group; PC, passive control group; *, for statistics concerning between-subjects factor effects, the table presents the standard error (SE) instead of the standard deviation (SD).

**Table 4:** Descriptive statistics for BCST, correct movements and TOL, ratio of movements performed to the minimum number of movements by measurement and group.

Group	Measurement	BCST: correct movements		TOL: Movement ratio Done/minimum number of moves	
		*M*	SD*	*M*	SD*
EG	P1	82.96	3.10	1.20	0.11
	P2	87.04	2.42	1.09	0.06
	Total	85.00	0.36	1.15	0.02
AC	P1	84.34	2.21	1.19	0.10
	P2	83.55	1.75	1.15	0.10
	Total	83.94	0.36	1.17	0.02
PC	P1	83.71	2.08	1.19	0.09
	P2	83.65	2.09	1.15	0.06
	Total	83.68	0.36	1.17	0.02
Total	P1	83.67	2.54	1.19	0.10
	P2	84.75	2.65	1.13	0.08

P, measurement; EG, experimental group; AC, active control group; PC, passive control group; *, for statistics concerning between-subjects factor effects, the table presents the standard error (SE) instead of the standard deviation (SD).

Preliminary analyses confirmed that the groups were equivalent in terms of age and sex (*p* > 0.05). No significant differences were observed between the groups at baseline in any of the memory tasks, suggesting comparability before the intervention.

The analysis showed that the within-subject effects of measurement were statistically significant for almost every variable related to memory, except for backward digit repetition. This means that differences were recorded between the pre- and post-stimulation measurements for the entire study sample. Regarding the number of correct forward digit repetitions and percentage of correctly performed movements in the BCST, higher values were recorded in the second measurement than in the first measurement. In BCST the effects were strong. On the other hand, the opposite is the case with the ratio of movements performed to the minimum number of movements in the TOL: after stimulation, the test result is lower than before stimulation. The effects were of moderate strength.

For the between-subjects factor, statistically significant effects were observed for the number of correct forward and backward digit repetitions and the percentage of correctly performed movements in the BCST. In the next step, *post-hoc* comparisons were performed with Bonferroni correction. In each case discussed, the PC group was characterized by a lower average value of both test measurements than the GE. These effects were of moderate strength.

Furthermore, the measurement group interaction effects were also significant for every memory task, except for the number of correct backward digit repetitions. Bonferroni-corrected *post hoc* tests were then re-run to test for precise differences between the groups across the measures. Comparisons between groups showed that for the number of correct digit repetitions forward, the percentage of correct movements in the BCST, the experimental group had higher test scores than the passive and active control groups, but only in the second measurement, and for the ratio of movements performed to the minimum number of movements in the TOL, the experimental group had lower test scores than the passive and active control groups, also only in the second measurement. No other differences were noted between the groups. Differences within groups between measurements were then assessed. The analysis showed that in the GE and AC groups there was an increase in the results of the task: forward digit repetition. Only the GE showed an increase in the percentage of correct movements on the BCST. Furthermore, a decrease in the ratio of completed movements to the minimum number of movements in the TOL was observed for each group. For forward digit repetition, the effects were moderate, whereas the other effects were strong.

## Discussion

Memory is a complex and multidimensional cognitive area consisting of clearly differentiated systems. As knowledge of memory increases, the number of concepts associated with it increases. Nowadays, memory is viewed as a set of integrated systems rather than as a single system (Bowman and Zeithamova, 2018; Kolb and Whishaw, 2003; Pąchalska et al., 2020).

Memory and its processes, i.e., coding, storage, retrieval, constitute the basis of the learning process, which results in relatively permanent changes in behavior. This is possible thanks to the phenomenon of brain (Longstaff, 2006; Jagodzińska, 2012; Lezak, 2012; Herzyk, 2015; Wiłkość, 2015) plasticity. Memory is key to the proper functioning of human beings. The area of the prefrontal cortex associated with it is equipped with neurons that are characterized by the ability to maintain activation for a longer period of time – both during the duration of a stimulus or event, and long after the interaction (Pąchalska et al., 2021; Riley and Constantinidis, 2016) has ended. This area of the brain is involved in storing representations of stimuli or actions over time. This means that information about external or internal stimuli and planned actions can be stored for some time because of the activity of this part of the brain. Through its ability to store and manipulate information, the DLPFC enables us to maintain goals in the working memory and take appropriate actions in accordance with established goals. Therefore during the performance of activities, this area can act as a “warehouse” of information necessary to achieve goals (Martin et al., 2021). As a result, the DLPFC plays a key role in maintaining coherence and direction of actions, as well as integrating the information needed to successfully achieve long-term goals.

Previous research has indicated that prefrontal cortex activity plays a pivotal role in maintaining information in memory, and its function extends beyond working memory alone (Riley and Constantinidis, 2016). The prefrontal cortex is engaged in the active processing of temporarily stored information, which is crucial for various cognitive functions including planning, decision-making, and communication (Funahashi, 2017; Stokes et al., 2013). The present study demonstrated that active transcranial direct current stimulation (tDCS) significantly enhanced memory across all evaluated indicators, except for performance on the Digit Span Backward task, which is considered a core measure of working memory the analysis of the memory span indices confirmed that tDCS enhanced the memory span.

In this study, participants who received tDCS exhibited superior performance on the Digit Span Forward (DSF) task, a measure of auditory-verbal memory capacity, compared with those in the other groups. The discrepancy emerged only in the second measurement, suggesting that the observed enhancement was attributable to the stimulation itself rather than to other factors such as placebo effects or practice effects. However, the absence of significant improvement in the Digit Span Backward task—widely regarded as a more direct and demanding measure of working memory—indicates that the observed enhancement should not be interpreted as a direct improvement in core working memory capacity. Rather, the findings point to improvements in short-term memory and higher-order cognitive processes that are functionally related to working memory, including attention, planning, and cognitive flexibility. This result is inconsistent with previous findings. In Jeon and Han's (2012) study, no discrepancy was observed in digit string recall capacity, i.e., auditory-verbal memory span. The study cited above produced analogous results for anodal and placebo stimulation, with both the left and right prefrontal cortices stimulated. One proposed explanation for the absence of observed effects in Jeon and Han (2012) study was that the maximum auditory-verbal memory span of nine items was insufficient to detect performance differences among healthy young adults.

However, in this study, the maximum memory span also included nine items. Expanding the task to include more items to be remembered would certainly increase the sensitivity of the tool. At the same time, it is worth noting the difference between Jeon and Han (2012) study and our study which may affect the results and interpretation of the stimulation effects. First, different stimulation parameters were used: the size of the electrodes (35 cm 2 in the cited study vs. 25 cm 2 in our own study), the current intensities administered (1 mA vs. 2 mA in our own study), and the stimulation time (20 min vs. 30 min). This finding may have important implications for our results. It can be suspected that the use of smaller electrodes while administering higher current allowed more precise and stronger stimulation of the area responsible for memory. In addition, the extended stimulation time allowed the increased neuronal activity in the stimulated brain area to last longer.

The experimental group exhibited a significant improvement in their ability to retain and adapt to changing rules, with the observed effects being of moderate strength. Participants who received tDCS stimulation demonstrated superior performance in tasks requiring flexible adaptation to novel rules, which is associated with enhanced cognitive efficiency. tDCS stimulation facilitated the rapid acquisition of new rules, their integration with pre-existing information in working memory, and their effective application in task execution. The observed improvement in cognitive flexibility suggests an enhancement in working memory efficiency. Following the stimulation cycle, participants in the experimental group demonstrated increased proficiency in retaining and manipulating information essential for goal attainment, as well as in maintaining coherence in their actions over time. The findings indicate that greater adaptability to novel conditions corresponds with more effective working memory functioning. These results are particularly noteworthy given the age of the participants who were in early adulthood. The improvement in cognitive flexibility may have significant implications for both professional and social domains. The ability to efficiently process information and navigate decision-making processes is crucial across various fields, and the observed cognitive enhancements may contribute to increased overall efficiency in these areas.

A particularly noteworthy finding is the percentage of correctly executed moves in the BCST, as a significant improvement in this metric was exclusively observed in the experimental group. Furthermore, the effect size was notably strong. tDCS contributed to enhanced memory efficiency, particularly in the retention and adaptation to changing rules. Following the stimulation cycle, participants exhibited improved maintenance of current rules in memory and applied this information more effectively in decision-making compared to their pre-stimulation performance. This can be interpreted as an increase in cognitive flexibility, encompassing the ability to adapt to new conditions, monitor rule changes, maintain them in working memory, and efficiently utilize them in decision-making processes. In all study groups, the second measurement revealed a decrease in the ratio of moves executed to the minimum number of moves required in the TOL, compared to the first measurement. This decline is likely attributable to the influence of a practice effect, as prior experience and task familiarity contributed to the development of more efficient problem-solving strategies in the TOL. However, when considering only the second measurement and comparing results across groups, a significantly lower ratio of executed moves to the minimum required moves was observed exclusively in the experimental group. A lower score in this metric is indicative of superior cognitive functioning, suggesting that tDCS facilitated improvements in memory retention, working memory maintenance, and decision-making processes based on strategic planning.

Memory processes encompass multiple functions, including encoding, maintenance, recall, and recognition (Wang et al., 2018). In the present study, participants did not increase the number of correctly repeated digits in the backward digit span task. This indicates that no significant improvement was observed in processes related to information manipulation in memory or the ability to reorganize newly acquired information within the auditory-verbal modality. No significant differences were found between the two measurements for the entire sample; however, it was noted that individuals who did not receive stimulation exhibited a lower mean score across both measurements compared to those who underwent tDCS. The effect size observed was of moderate strength.

It is important to emphasize that these results pertain exclusively to the auditory-verbal modality, and the measured indicator is associated with more complex processes than simple memory span. Similar conclusions were drawn by other researchers, who found that different variants of tDCS did not enhance performance in the auditory-verbal memory modality (Teo et al., 2011; Wang et al., 2018). Wang and colleagues (2018) reported no significant difference between the active and placebo groups when applying anodal (i.e., excitatory) stimulation. However, both groups exhibited a statistical trend toward improvement in the examined domain. In contrast, such an effect was not observed with cathodal (i.e., inhibitory) stimulation (Teo et al., 2011; Wang et al., 2018). Given that repeated memory measurements often reveal a tendency for performance improvement, it is plausible that a practice effect, or learning effect, was at play (Wang et al., 2018).

The backward digit span task, in which participants did not demonstrate improved performance, engages both attentional processes and the ability to generate sequences in reverse order. However, these indicators should not be considered a general measure of memory (Golden, 1979; Hornowska, 2004). Performance in this task may be influenced by situational distractors or emotional tension (Kucharska-Pietura et al., 2012). Nevertheless, attributing the lack of improvement solely to these factors seems unlikely, as they would have needed to exert an effect during both measurements and be strong enough to completely counteract the stimulation effect.

A more plausible explanation lies in the differences between n-back tasks in the auditory-verbal modality (Au et al., 2016; Friehs and Frings, 2019; Karthikeyan et al., 2021; Ruf et al., 2017), which are commonly used to assess working memory - including in the context of tDCS - and digit span tasks. In n-back tasks, participants must recognize repetitions within a sequentially presented series of stimuli, but only at a specific position, such as the second (2-back) or third (3-back) position from the end of the sequence. In addition to maintaining a set of presented stimuli in memory, participants must continuously update this set with each new stimulus, as the item at the n-back position shifts with every subsequent presentation (Orzechowski, 2012).

In contrast, digit span tasks require participants to generate or recall previous information through free recall, whereas n-back tasks rely on recognition-based retrieval, in which participants select previously presented stimuli. It is possible that tDCS enhances memory performance more effectively when rapid recognition is required rather than free recall (Redick and Lindsey, 2013). The findings of the present study appear to support this hypothesis. In summary, the obtained data partially confirmed earlier findings suggesting that stimulation of the left dorsolateral prefrontal cortex (DLPFC) enhances memory functioning in both healthy and clinical populations. Some of the earliest studies on cognitive enhancement using tDCS, conducted by Fregni et al. (2005), demonstrated that anodal stimulation of the left DLPFC significantly improves response accuracy during active stimulation compared to the placebo group. However, these findings were exclusively observed in response to anodal stimulation, rather than cathodal stimulation. These results provide further evidence to support the hypothesis that enhancing cortical excitability in the DLPFC may improve memory performance. Anodal tDCS is a method of electrically stimulating the brain by applying a direct current of positive charge to the scalp over the targeted region, in this case the DLPFC. This current exerts an influence on neuronal activity within the area being stimulated. The application of anodal tDCS has been demonstrated to augment neuronal excitability within the DLPFC by means of depolarising cell membranes. Depolarisation is defined as an increase in the electrical potential difference across the cell membrane, thereby increasing the likelihood of triggering an action potential and facilitating the transmission of signals between neurons. Consequently, this process results in heightened neuronal activity in the DLPFC.

Additionally, anodal tDCS may influence synaptic plasticity, which refers to the ability of synapses to modify their connection strength. This stimulation can enhance the activity of N-methyl-D-aspartate (NMDA) receptors, which play a crucial role in signal transmission between neurons as well as in synaptic plasticity, learning, and memory processes. Increased NMDA receptor activity leads to an elevated influx of calcium ions into neurons, thereby initiating synaptic plasticity mechanisms (Polanowska and Seniów, 2010). Consequently, this may improve the functioning of neural networks associated with memory.

Numerous subsequent studies have examined factors such as electrode placement, current density, and stimulation duration, all of which can impact the efficacy of tDCS (Ke et al., 2019). These studies have confirmed the effectiveness of anodal DLPFC stimulation in enhancing memory performance, including working memory (Au et al., 2016; Boehringer et al., 2013; Coffman et al., 2014; Gözenman and Berryhill, 2016; Hill et al., 2016; Richmond et al., 2014; Ruf et al., 2017; Talsma et al., 2017).

Neuroimaging studies using electroencephalography (EEG) and functional near-infrared spectroscopy (fNIRS) provide evidence that tDCS can alter brain activity (Bogaard et al., 2019; Jang et al., 2019; Jones et al., 2017). Some researchers suggest that anodal stimulation of the left dorsolateral prefrontal cortex (DLPFC) may selectively enhance performance in cognitively demanding tasks. This effect is attributed to the focal improvement of executive functions and/or cognitive abilities under conditions of high task difficulty, rather than a general increase in arousal and/or alertness (Pope et al., 2015). This hypothesis is partially supported by the findings of the present study.

Several limitations of this study should be acknowledged. First, although the sample size (*N* = 90) was adequate for initial exploratory analysis, it remains relatively small in the context of tDCS research, where individual variability in neuroanatomy can significantly impact outcomes. Second, the study lacked a long-term follow-up phase, limiting conclusions about the persistence of the observed effects. Third, while the inclusion of both active and passive control groups strengthens internal validity, the subjective experience of stimulation (e.g., tingling, warmth) was not systematically measured. This omission constitutes a methodological limitation, as it precludes the evaluation of blinding effectiveness and the potential influence of expectation effects. Although anecdotal reports indicated that most participants did not perceive clear differences between conditions, a standardized post-session assessment would have been preferable and should be implemented in future research. Standardized self-report measures would address this issue. Fourth, an imbalance in educational attainment between groups may have functioned as a confounding variable influencing cognitive outcomes. Although the distribution did not indicate substantial disparities in functional cognitive capacity, we chose not to statistically control for this variable (e.g., through ANCOVA). As such, this limitation should be considered when interpreting between-group differences. Future research should employ stratified randomization or incorporate covariate adjustment to address such imbalances more rigorously. Furthermore, physiological variables, such as quantitative electroencephalography (qEEG), were not controlled, which could have provided additional insights into the effects of tDCS on brain activity. Finally, improvements observed in both experimental and control conditions suggest the possible influence of placebo effects or general test–retest learning, which should be further investigated.

## Conclusion

1. Transcranial direct current stimulation (tDCS) in the anode F3, cathode Fp2 configuration has a beneficial effect on the improvement of executive functions in healthy young adults. This includes memory-related aspects such as auditory-verbal memory span, the ability to maintain an intended plan in memory, as well as the capacity to retain and coordinate different task components, enabling the effective execution of information maintenance processes. However, since the Digit Span Backward task did not show significant improvement, the findings should be interpreted as evidence of improved short-term memory and working-memory-related executive functions, rather than a direct enhancement of core working memory capacity.
2. tDCS stimulation is an effective method for enhancing cognitive functioning in healthy individuals during early adulthood.
3. Further research is necessary to assess the long-term effects of tDCS and to elucidate the precise mechanisms underlying its effects.

## Data Availability

The raw data supporting the conclusions of this article will be made available by the authors, without undue reservation.
